# The diversity of the fecal bacterial community and its relationship with the concentration of volatile fatty acids in the feces during subacute rumen acidosis in dairy cows

**DOI:** 10.1186/1746-6148-8-237

**Published:** 2012-12-06

**Authors:** Shengyong Mao, Ruiyang Zhang, Dongsheng Wang, Weiyun Zhu

**Affiliations:** 1 College of Animal Science and Technology, Nanjing Agricultural University, Nanjing, 210095, China

**Keywords:** Subacute rumen acidosis, Fecal bacterial community, Volatile fatty acid, Dairy cows

## Abstract

**Background:**

Sub-acute ruminal acidosis (SARA) is a well-recognized digestive disorder found in particular in well-managed dairy herds. SARA can result in increased flow of fermentable substrates to the hindgut, which can increase the production of volatile fatty acids, alter the structure of the microbial community, and have a negative effect on animal health and productivity. However, little is known about changes in the structure of the microbial community and its relationship with fatty acids during SARA. Four cannulated primiparous (60 to 90 day in milk) Holstein dairy cows were assigned to two diets in a 2 × 2 crossover experimental design. The diets contained (on a dry matter basis): 40% (control diet, COD) and 70% (SARA induction diet, SAID) concentrate feeds. Samples of ruminal fluid and feces were collected on day 12, 15, 17 and 21 of the treatment period, and the pH was measured in the ruminal and fecal samples; the fecal microbiota was determined by pyrosequencing analysis of the V1–V3 region of amplified 16S ribosomal RNA (16S rRNA).

**Results:**

SAID decreased ruminal and fecal pH and increased the propionate, butyrate and total volatile fatty acid (TVFA) concentration in feces when compared with the COD. A barcoded DNA pyrosequencing method was used to generate 2116 16S operational taxonomic units (OTUs). A total of 11 phyla were observed, distributed amongst all cattle on both diets; however, only 5 phyla were observed in all animals regardless of dietary treatment, and considerable animal to animal variation was revealed. The average abundance and its range of the 5 phyla were as follows: Firmicutes (63.7%, 29.1–84.1%), Proteobacteria (18.3%, 3.4–46.9%), Actinobacteria (6.8%, 0.4–39.9%), Bacteroidetes (7.6%, 2.2–17.7%) and Tenericutes (1.6%, 0.3–3%). Feeding the SAID resulted in significant shifts in the structure of the fecal microbial community when compared with the traditional COD. Among the 2116 OTUs detected in the present study, 88 OTUs were affected significantly by diet; and the proportion of these OTUs was 20.6% and 17.4% among the total number of sequences, respectively. Among the OTUs affected, the predominant species, including OTU2140 (G: *Turicibacter*), OTU1695 (G: *Stenotrophomona*s) and OTU8143 (F: Lachnospiraceae), were increased, while the abundance of OTU1266 (S: *Solibacillus silvestris*) and OTU2022 (G: *Lysinibacillus*) was reduced in the SAID group compared with the COD. Further, our results indicated that the fecal volatile fatty acid (VFA) concentrations were significantly related to presence of some certain species of Bacteroidete*s* and Firmicute*s* in the feces.

**Conclusions:**

This is, to our knowledge, the first study that has used barcoded DNA pyrosequencing to survey the fecal microbiome of dairy cattle during SARA. Our results suggest that particular bacteria and their metabolites in the feces appear to contribute to differences in host health between those given SAID and traditional COD feeding. A better understanding of these microbial populations will allow for improved nutrient management and increased animal growth performance.

## Background

Subacute ruminal acidosis (SARA), also known as chronic or sub-clinical acidosis, is a common health problem in many dairy herds [1]. Results from field studies indicate a high prevalence of SARA in high-producing dairy herds of some regions as producers respond to the demand for increased milk production with diets containing a higher proportion of grain [2]. Dairy herds that experience SARA have a decreased efficiency of milk production, impaired cow health, and high rates of involuntary culling [3]. A previous study showed that, in cases of SARA, the escape of large amounts of undigested feed from the rumen and small intestine can result in extensive fermentation in the hindgut during grain-induced SARA [4]. This fermentation results in increased acidity of the hindgut contents and feces. The increased acidity may result in damage to and sloughing of the epithelial cells in the large intestine. Damage to the large intestine and increased concentrations of organic acids in the gut lumen may play a role in causing the diarrhea often seen with ruminal acidosis [5].

In the hindgut of bovine species, the microbiota, including bacteria, protozoa and fungi, possess cellulase, protease, deaminase, and urease activities, and the products of fermentation include volatile fatty acid (VFA), ammonia nitrogen, and microbial cells [6]. As mentioned above, the increased amounts of carbohydrate in the large intestine during SARA can stimulate fermentation by these bacteria and increase the acidity of the hindgut contents and feces; this indicates that the microbial community of the hindgut may be altered during SARA. Further, some studies have also shown that numbers of enterohemorrhagic *E. coli* are higher in the feces of grain-fed cattle. In turn, when cattle were abruptly switched from a high corn diet to a forage diet, generic *E. coli* populations declined 1000-fold within 5 days [7,8]. In general, all these studies suggest that high grain feeding will lead to the production of more volatile fatty acids (acetate, butyrate, and propionate) and a change in the structure of the microbial community in the hindgut, which will result in a relative increase in the number of some pathogens during SARA. However, there is a lack of published studies investigating changes in the structure of the hindgut microbial community during SARA in dairy cows. Therefore, the main purpose of this study was to investigate the diversity of the bacterial community and to evaluate its relationship with the concentration of short volatile fatty acid (VFAs) in the feces during SARA in dairy cows.

## Results

### Effects of SAID feeding on ruminal pH and VFA concentration in feces

When compared with feeding of the COD, the fecal pH was significantly lower and the propionate, butyrate, and TVFA concentrations were significantly higher in the SAID group (Table 1). There were no significant differences in valerate, isobutyrate, isovalerate, or the ratio of acetate to propionate between the two diets. A tendency towards increased fecal acetate production was observed in the SAID group when compared with the COD.

**Table 1:** Fecal pH and VFA concentration (n=4)

**Parameter**	**COD**	**SAID**	**Std. error**^**a**^	**P value**	**95% confidence interval**^**b**^	
					**Lower**	**Upper**
Fecal pH	7.15	6.42	0.108	0.041	6.34	7.10
Acetate, mM	46.49	63.12	4.545	0.061	42.18	67.42
Propionate, mM	10.36	13.90	0.399	0.003	11.02	13.24
Butyrate, mM	6.86	17.35	0.729	0.001	10.08	14.13
Valerate, mM	0.69	0.46	0.093	0.161	0.32	0.83
Isobutyrate, mM	1.56	1.94	0.470	0.595	0.45	3.05
Isovalerate, mM	0.51	0.65	0.223	0.662	−0.04	1.20
Total VFA, mM	66.46	97.56	4.989	0.012	68.16	95.86

^a^Std. Error of the mean for the SAID group.

^b^95% CI for the SAID group.

**Figure 1:**
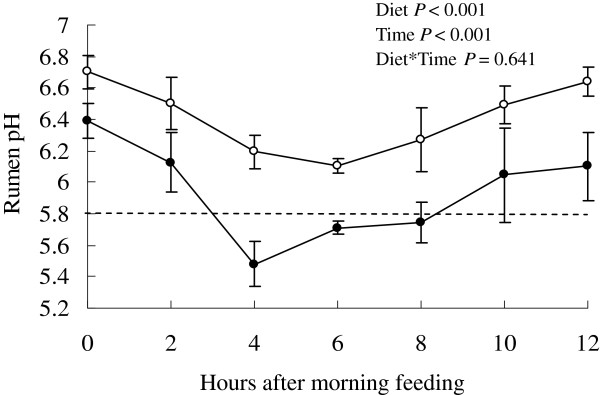
**Diurnal variation of rumen pH in lactating Holstein cows fed with COD (○) or SAID (***●***).** Means ± standard deviation; n=4.

In this study, diurnal data indicated that cows fed the COD had greater ruminal pH when compared with the SAID group (*P*<0.001) (Figure 1). Ruminal pH was also affected by sampling hour (*P*<0.001). In general, the ruminal pH declined in all cows, regardless of the diet used, during the first few hours after the morning feeding. However, during the time period from almost 3 h after the feeding until the sampling at 8 h, the average ruminal pH was below 5.8 in the SAID group and remained significantly lower than that of the COD cows; the duration for which the ruminal pH was less than 5.8 was about 5.1 hours after the first feeding in the SAID group.

### General DNA sequencing observations

**Figure 2:**
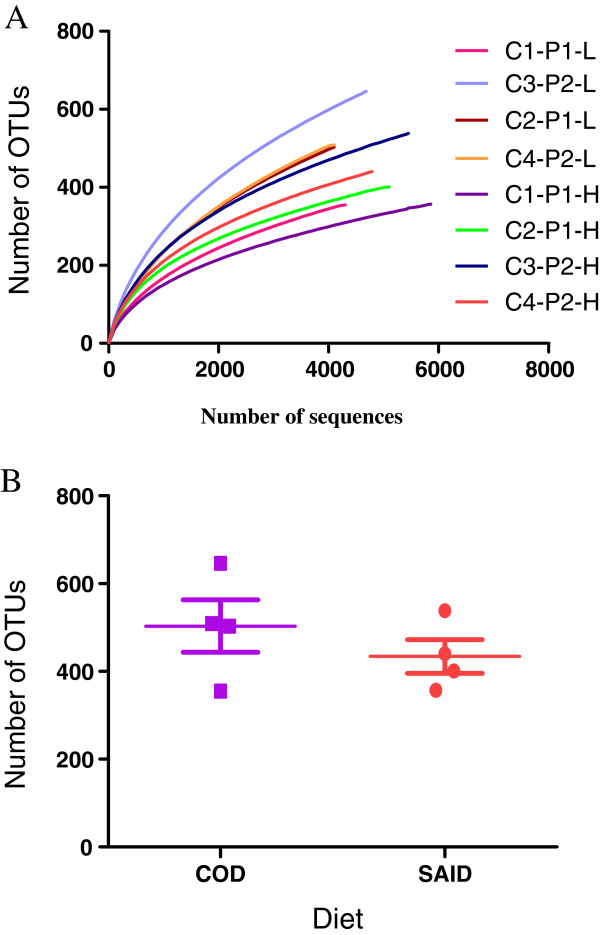
**Summary of diversity assessments based on operational taxonomic unit (OTUs) (3% divergence) for each sample. A**. Summary of rarefaction results based on operational taxonomic unit (OTUs) (3% divergence) for each sample. Rarefaction curves are displayed for each of the samples. COD: C1-P1-L, C2-P1-L, C3-P2-L, C4-P2-L. SAID: C1-P2-H, C2-P2-H, C3-P1-H, C4-P1-H. **B**. Summary of OTUs for each dietary treatment. Dots indicate the OTUs associated with each animal. COD = Control diet, SAID = SARA induction diet.

The total number of high quality 16S OTUs recovered from each animal is listed in Table 2. The average number of OTUs returned for each diet was: COD, 503; SAID, 414. Rarefaction curves indicated that there was a high level of microbial diversity in dietary treatments (Figure 2A). The total abundance observed for OTUs in the COD and SAID groups are indicated in box plots (Figure 2B), and a substantial animal-to-animal variation was observed in both the COD and SAID groups.

**Table 2:** Distribution of 16S OTUs

**Treatment**	**Aminal ID**	**No 16S OTUs**
COD	C1-P1-L	355
COD	C2-P1-L	503
COD	C3-P2-L	646
COD	C4-P2-L	509
SAID	C1-P1-H	357
SAID	C2-P1-H	401
SAID	C3-P2-H	538
SAID	C4-P2-H	440

The dietary treatment, animal ID, and no. of OTUs obtained per fecal sample from each animal.

**Figure 3:**
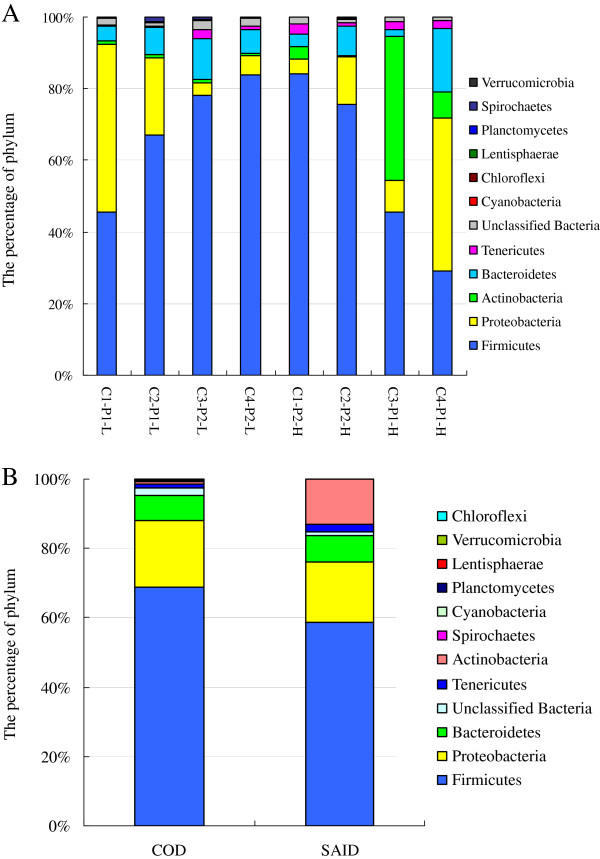
**Distributions of phyla. A.** The distribution of phyla for each sample. **B**. Distribution of the phyla averaged across the dietary treatments. Legend as for Figure 2 .

A total of 11 phyla were observed, distributed amongst all the cattle on the two diets (Figure 3). These are listed in order of average abundance and with their respective ranges: Firmicutes (63.7%, 29.1–84.1%), Proteobacteria (18.3%, 3.4–46.9%), Actinobacteria (6.8%, 0.4–39.9%), Bacteroidetes (7.6%, 2.2–17.7%), Tenericutes (1.6%, 0.3–3%), Cyanobacteria (0.08%, 0.0–0.23%%), Spirochaetes (0.3%, 0.0–1.1%), Lentisphaerae (0.05%, 0.0–0.17%), Planctomycetes (0.06%, 0.0–0.17%), Chloroflexi (0.01%, 0.0–0.04%), and Verrucomicrobia (1.43%, 0.0–23.6%). Greater than 98.4% of the total bacterial abundance was observed in these 11 phyla, with the remaining abundance represented by unclassified bacteria.

At the genus level, overall, the sequences were assigned to 154 different genera. Of these, the COD and SAID groups were represented by 105 and 124 genera, respectively (Table 3). Genera were present across the entire sample collection in the COD or the SAID group were defined as common, there were 41 and 31 common bacterial genera in cows fed the two diets, receptively.

**Table 3:** Number of bacterial genera identified at the genus level to be common in COD or SAID

**Parameters**	**COD**	**SAID**
Total ^a^	105	124
Common genera ^b^	41	31

^a^ The total number of genera identified in the two diets, respectively.

^b^ The number of genera that are common in COD or SAID.

### Influence of SAID feeding on bacterial phyla, classes, orders and families of fecal microbiota

When compared with COD feeding (Figure 3B), SAID feeding decreased the percentage of Cyanobacteria (*P*=0.014). However, no significant differences were observed between the dietary treatments in the abundance of Actinobacteria (*P*=0.222), Bacteroidetes (*P*=0.935), Chloroflexi (*P*=0.374), Firmicutes (*P*=0.237), Lentisphaerae (*P*=0.250), Planctomycetes (*P*=0.188), Proteobacteria (*P*=0.865), Spirochaetes (*P*=0.260), Tenericutes (*P*=0.150), or Verrucomicrobia (*P*=0.246).

The response of the most abundant bacteria at the phylogenetic levels of class, order and family is revealed in a series of heat maps (Additional file 1: Figure S1A, Additional file 2: Figure S2A, Additional file 3: Figure S3A). At the class level, the class Clostridia, Bacilli, Bacteroidia, Erysipelotrichi, and Gammaproteobacteria were the most abundant in all the animals (Additional file 1: Figure S1A). The relative abundance of Bacilli (*P*=0.008), unclassified Firmicutes (*P*=0.010), and unclassified Cyanobacteria (*P*<0.001) was significantly lower in the SAID group when compared with COD (Additional file 1: Figure S1B). At the level of order, the predominant orders were: Clostridiales, Bacillales, Enterobacteriales, Bacteroidales, Erysipelotrichales, and Lactobacillales (Additional file 2: Figure S2A), and the percentage of Bacillales (*P*=0.028), unclassified Firmicutes (*P*=0.010), unclassified Cyanobacteria (*P*<0.001) and Rhodospirillales (*P*<0.025) was decreased in the SAID group, while the order Xanthomonadales (*P*<0.013) was increased in percentage in the SAID group (Additional file 2: Figure S2B) when compared with the COD. At the family level, the predominant families included Planococcaceae, Enterobacteriaceae, Moraxellaceae, Peptostreptococcaceae, Lachnospiraceae, Ruminococcaceae, and Erysipelotrichaceae (Additional file 3: Figure S3A). As compared with the feeding of COD, the percentages of unclassified Pseudomonadales (*P*<0.035), Rhodospirillaceae (*P*<0.001), unclassified Bacillales (*P*<0.001), unclassified Cyanobacteria (*P*<0.001), Streptococcaceae (*P*<0.001*)*, unclassified Firmicutes (*P*<0.010), and Planococcaceae (*P*=0.007) were lower, while the abundance of Xanthomonadaceae (*P*<0.013) was higher in the SAID group (Additional file 3: Figure S3B).

### Influence of SAID feeding on the genera of fecal microbiota

**Figure 4:**
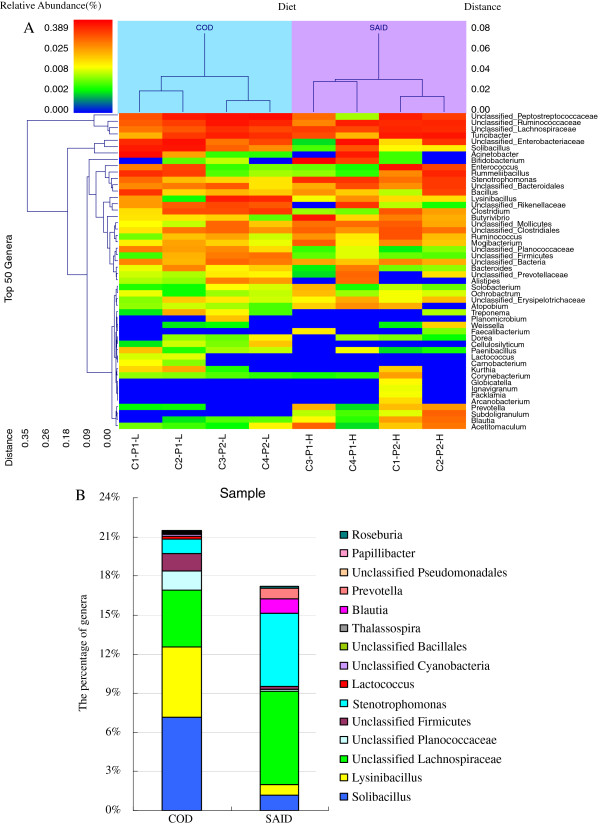
**Influence of SAID on fecal microbiota of dairy cattle for the top 50 most abundant genera.** Legend as for Figure 2 .

The influence of the SAID on the fecal microbiome was observed from double hierarchical cluster analysis on the top 50 most abundant genera (≥ 97% of the total number of bacterial genera observed) and clustered by dietary treatment (Figure 4A). Among these genera, unclassified Peptostreptococcaceae, unclassified Ruminococcaceae, unclassified Lachnospiraceae and *Turicibacter* occurred together in one cluster, whereas unclassified Enterobacteriaceae, *Solibacillus*, and *Acinetobacter* resided in the next most distant cluster. The other 43 genera cohabited in another main cluster. The average abundance by treatment of the genera and the response of the taxa to diet, i.e. influenced by (*P*<0.10) or significantly associated with (*P*<0.05) diet, are presented in Figure 4B. In general, when compared with the COD group, SAID feeding increased the percentage of unclassified Lachnospiraceae (*P*=0.022), *Stenotrophomonas* (*P*=0.013), *Blautia* (*P*=0.034) and *Prevotella* (*P*=0.037), while the percentages of *Solibacillus* (*P*=0.005), *Lysinibacillus* (*P*=0.001), unclassified Planococcaceae (*P*=0.015), unclassified Firmicutes (*P*=0.010), *Lactococcus* (*P*<0.001), unclassified Cyanobacteria (*P*<0.001), unclassified Bacillales (*P*=0.001), *Thalassospira* (*P*<0.001), unclassified Pseudomonadale (*P*=0.035), *Papillibacter* (*P*=0.020), and *Roseburia* (*P*=0.006) were lower in the SAID group. A tendency for a decrease in unclassified Peptostreptococcaceae (*P*=0.095) and *Clostridium* (*P*=0.088) was observed in the SAID group, and there was a tendency for an increase in the percentage of *Succinivibrio* (*P*=0.072).

### Influence of SAID feeding on fecal microbiota – OTUs

For OTUs at 3% distance (species level), 2116 different phylotypes were detected among all the samples. Of these, 1349 phylotypes were found in COD and 1207 in SAID samples. The percentage of species-level taxa shared by the COD and SAID groups was 21% (440 species) (Additional file 4: Figure S4). The mean number of OTUs at the 3% dissimilarity level present per COD sample was 503 (range, 355 to 646), compared with 434 OTUs (range, 357 to 538) in the SAID samples. Data on the shared genera are available as supporting information (Additional file 5: Table S1).

**Figure 5:**
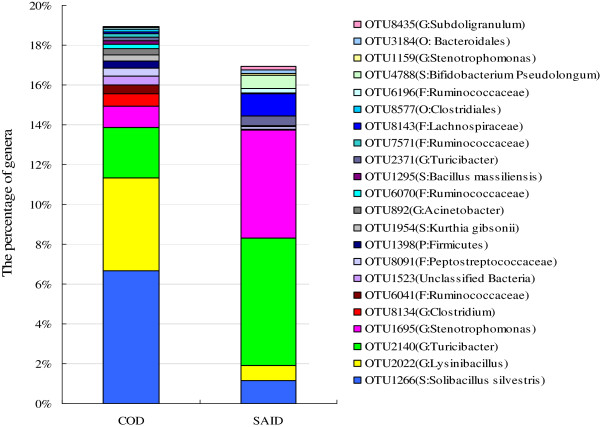
**Influence of SAID on fecal microbiota of dairy cattle at species level.** Only the predominant OTUs that were significantly affected in percentage by diet are presented. COD = Control diet, SAID = SARA induction diet.

Among the 2116 OTUs detected in the present study, 88 OTUs were significantly affected by diet, and the proportion of these OTUs was 20.63% and 17.41% among the total number of sequences (Additional file 6: Table S2), respectively. When compared with the COD, the predominant species, including OTU2140 (G: *Turicibacter*) (*P*=0.039), OTU1695 (G: *Stenotrophomonas*) (*P*=0.016), and OTU8143 (F: Lachnospiraceae) (*P*=0.019) were increased in percentage (Figure 5), while the abundance of OTU1266 (S: *Solibacillus silvestris*) (*P*=0.005) and OTU2022 (G: *Lysinibacillus*) (*P*=0.001) were reduced in the SAID group.

### Correlations between the fecal microbiota and the fecal VFA concentration

**Figure 6:**
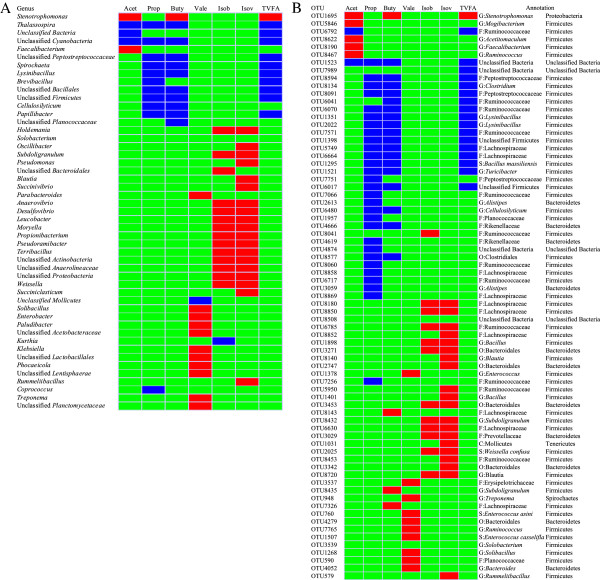
**Correlations between the fecal microbiota and the fecal VFA concentrations.** Only the genera or species for which abundance was significantly associated with the fecal VFA concentration are presented; the blue represents a negative correlation between the abundance of the species and the VFA concentration (*P*<0.05), the red color represents a positive correlation (*P*<0.05), and the green shows that the correlation was not significant (*P*>0.05). **A**: Correlations between the abundance of the genera and the fecal VFA concentration. **B**: Correlations between the abundance of the species and the fecal VFA concentration. Acet: =acetate; Prop: =propionate; Buty: =butyrate; Isob: =isobutyrate; Isov: =isovalerate; TVFA: =total volatile fatty acid. S: =species; G: =genera; F: =family; O: =order; C: =class.

Particular genera were found to be linked to VFA concentration as follows (Figure 6A): five genera were linked to acetate (positive correlation: two genera; negative correlation: three genera), 11 genera were correlated with propionate, 11 genera were associated with butyrate (positive correlation: one genus; negative correlation: 10 genera), 12 genera were correlated with valerate (positive correlation: 11 genera, negative correlation: one genus), 14 genera were correlated with isobutyrate (positive correlation: 13 genera, negative correlation: one genus), 19 genera were associated with isovalerate, 11 genera were linked with TVFA (positive correlation: one genus, negative correlation: 10 genera).

At the species level, the species were found to be linked to VFA concentration as follows (Figure 6B): seven OTUs were linked to acetate (positive correlation: five genera; negative correlation: two genera), 28 OTUs were correlated with propionate, 22 OTUs were associated with butyrate (positive correlation: four genera; negative correlation: 18 genera), seven genera were correlated with valerate, 11 genera were correlated with isobutyrate, 19 genera were associated with isovalerate, 18 genera were correlated with TVFA (positive correlation: one genus; negative correlation: 17 genera). Most of these species belong to the phyla Bacteroidetes and Firmicutes.

## Discussion

### Effects of SAID feeding on ruminal pH

The presence of SARA is a major concern in terms of both productivity and animal welfare. Ruminal pH thresholds of 5.8 or less are generally used to give a clinical diagnosis of SARA [9,10]. In the present study, the duration for which the ruminal pH was less than 5.8 was about 5.1 hours in the SAID group after the first feed; therefore, we considered that SARA occurred in the cattle fed with SAID.

### General characteristics of bovine fecal microbial communities

Deep sequencing of the fecal samples collected from the dairy cows fed with the two diets provided a detailed view of the cattle fecal microbiome in the samples tested. We detected members of 11 phyla of bacteria. The majority of the pyrotags belonged to the Firmicutes, Proteobacteria, Bacteroidetes, Actinobacteria, and Tenericutes These phyla have been shown previously to constitute the majority of gut-associated phylotypes in a variety of different mammalian species [11-13], which suggests that these phyla, especially the Firmicutes, play a critical role in the microbial ecology of the mammalian gut, including the bovine gut. Other phyla represented were the Chloroflesi, Lentisphaerae, Planctomycetes, Spirochaetes, and Verrucomicrogia. This indicates that, even though all of the bacterial phyla contain a diverse range of taxa, the metabolic potential of some phyla most likely allows some to dominate in bovine feces while others remain less abundant.

A comparison of all the genera from this study identified only 41 and 31 genera that were present across the entire sample collection in the COD or the SAID group, respectively (Table 3). The majority of these genera were classified as Firmicutes and Proteobacteria. In addition, the characterization of the percentage abundance of all taxa in cattle given the two diets (Figure 4B) suggests that the population structure of the microorganisms, including those of the genera *Solibacillus*, *Lysinibacillus*, unclassified Lachnospiraceae, *Stenotrophomonas*, *Blautia*, and *Prevotella*, were dramatically different in the COD and the SAID group. Indeed, many Lachnospiraceae, *Blautia*, and *Prevotella* spp. are common inhabitants of the gastrointestinal tracts and feces of cattle and goats [14-16]. The shifts in the abundance of these microorganisms in the feces of the dairy cows in this study suggest that members of these taxa harbor the metabolic potential to thrive when the diet is transferred rapidly from a traditional COD to a high-concentrate diet.

### Effects of SAID feeding on fecal microbiota

The SAID evaluated in this study seemed to have a complex effect on the fecal microbiota, and a common set of taxa seem to be responsive to the influence of SAID vs. traditional COD. Some of these taxa have been identified in other studies to be responsive to or seemingly influenced by a high-grain diet, regardless of the differences in experimental protocols and animals (beef vs. dairy cattle) [17,18]. A more likely explanation for shifts in the microbial community structure in animals fed SAID may be the abundance and digestibility of starch in the SAID compared with that in the fibrous plant materials associated with the COD. The hindgut of cattle is ideally suited for the fermentation of sugars from fibrous plant materials [19]. Bovine species themselves do not produce the required fiber-degrading enzymes; instead, they harbor fungi, protozoa, and bacteria in their guts that can ferment the fiber. Thus, bovine digestive physiology is dictated largely by the presence of fibrous materials in the rumen. If such animals are fed fiber-deficient diets, such as a high-grain diet, normal digestive processes can be disrupted, leading to the accumulation of fermentation acids, and thus lowering the ruminal pH [20-22]. In turn, these changes in the sources of sugar and starch, combined with shifts in pH, alter the digestive habitat, resulting in a fecal bacterial community that can have an impact on VFA production and pathogen shedding [8,23]. In the present study, the concentration of starch in the feces from SAID-fed animals was 3 times higher than that in COD-fed cattle (4.43% vs 1.10%). Therefore, the large amount of starch in bovine feces during SAID feeding may be responsible for the changes in the composition of fecal microbial species. However, it should be noted that the SAID used in this study does not represent all types of SARA-inducing diet. A previous study showed that the rumen pH and fermentation variables obtained with wheat challenge were indicative of lactic acidosis, whereas butyric and propionic SARA was observed for corn and beet pulp challenges, respectively [24]. In the present study, the SARA was induced by a corn-based diet. Thus, further work is required to determine whether particular grain sources can influence the ecology of bovine fecal bacterial communities. Nonetheless, this study indicated that there were fundamental differences in the fecal microbial communities of animals in the COD and SAID groups.

Feeding high grain to cattle has a significant effect on the animal health [25]. Studies have indicated that varying the forage to grain ratio in cattle rations can have a marked effect on populations of *E. coli*. Some studies indicated that over feeding grain increased generic *E. coli* and/or O157:H7 populations [7,26-28]. Although *E. coli* O157:H7 was not detected in any of the present samples and *Streptococcus pluranimalium* and *Campylobacter* spp. (Additional file 5: Table S1) were only detected in one fecal sample from the dairy cows fed with the SAID, the data presented herein demonstrate that changes in rations can affect the microbial ecology of the intestinal tract of cattle, which could potentially affect food safety.

### The correlation between composition of the bacterial community and concentration of volatile fatty acids

This study showed the presence of high concentrations of propionate and butyrate in the feces of dairy cattle fed with the SAID. The concentrations of volatile fatty acids in this study are similar to results obtained in other studies [29]. Correlation analysis showed that a significant positive correlation was observed between the genus *Stenotrophomonas* and the levels of acetate, propionate, and TVFA (Figure 6). A previous study showed that *Stenotrophomonas* spp. were the predominant bacteria found in samples of feces from pigs and chickens and existed as opportunistic pathogens [30]. Our present study showed that SAID feeding was significantly associated with the percentage of the genus *Stenotrophomonas*, and TVFA production, and this indicates that SAID feeding of livestock may increase the risk of human infection with these opportunistic pathogens.

In the present study, genera including *Thalassospira*, unclassified Cyanobacteria, unclassified Peptostreptococcaceae, *Spirochaeta*, *Lysinibacillus*, unclassified Bacillales, and *Papillibacter* were negatively correlated with the production of propionate, butyrate and TVFA, respectively, and we also observed that *Thalassospira* and unclassified Cyanobacteria were negatively correlated with acetate concentration, which may have been caused by the toxic effect of this VFA. This hypothesis is supported by a proposed mechanism for fatty acid toxicity, in which short-chain organic acids, including acetate, propionate and butyrate, can diffuse freely across the bacterial membrane into the cell. Inside the bacterial cell, the acid dissociates, thereby reducing the internal pH, which will cause internal cell damage [31-34].

Branched-chain VFA(BCVFA) are derived from branched-chain amino acids such as leucine, valine, and isoleucine, and some studies have shown that several species of rumen bacteria, including *Prevotella ruminicola*, *Bacteroides ruminicola* and *Megasphaera elsdenii*, have a specific requirement for one or more of the BCVFA [35,36]. Allison and co-workers [37,38] have indicated the importance of isovaleric and isobutyric acids in the synthesis of amino acids and lipids in two species of *Ruminococcus* found in the rumen. Interestingly, our studies have shown that a group of genera, including *Holdemania*, *Subdoligranulum*, *Anaerovibrio*, *Desulfovibrio*, *Leucobacter*, *Moryella*, *Propionibacterium*, *Pseudoramibacter*, *Terribacillus*, unclassified Actinobacteria, unclassified Anaerolineaceae, unclassified Proteobacteria, and *Weissella*, are positively correlated with isobutyrate and isovalerate. Other genera, such as *Parabacteroides*, unclassified *Mollicutes*, *Solibacillus*, *Enterobacter*, *Paludibacter*, unclassified Acetobacteraceae, *Klebsiella*, unclassified Lactobacillales, *Phocaeicola*, unclassified *Lentisphaerae*, *Treponema*, and unclassified Planctomycetaceae, were positively associated with valerate. However, to date, we know very little about the nutritional characteristics of these bacteria, and it is uncertain whether the growth of these bacteria is stimulated by BCVFA; therefore, the mechanism behind the association between the abundance of these bacteria and the concentrations of BCVFA is still not clear. Nonetheless, this study indicates that the concentrations of isobutyrate and isovalerate may be associated with a particular group of bacteria, and that valerate concentration is linked with a different bacteria group.

In the present study, although we have shown there are possible associations between some VFA and the microbial community, our data are limited and may be biased, because the VFA and the microbial community measured in this study were collected at a single time point. On the other hand, the decrease or increase in these particular genera may be dependent on many other variables (e.g. competition for substrates or production of antimicrobial substances). Similarly, the production of VFA is dependent on other variables (e.g. bacterial metabolism) [21,39]. Therefore, it remains uncertain whether these significant correlations were influenced by other variables, and therefore if these significant correlation are causal. However, our results have revealed that the genera associated with propionate and butyrate producers may be similar, that the genus linked with the production of valerate is very distinctive, and that the species associated with isobutyate and isovalerate concentrations may belong to one group of ruminal bacteria.

## Conclusions

This study is the first to use a barcoded DNA pyrosequencing method to survey the fecal microbiome of dairy cattle during SARA. Our analyses indicated that the microbial community in the feces of dairy cattle was affected by SAID feeding, and that SAID feeding may increase the risk of human infection with some opportunistic pathogens. Our work also identified associations between fecal ecology and VFA. From the correlation analysis, genera representing some specific groups of bacteria were shown to be associated with some VFA. A better understanding of these microbial populations will allow for improved nutrient management and increased animal growth performance.

## Methods

### Ethics statement

All animal studies followed the regulations of the review committee of laboratory animal welfare and ethics and the protocol of the review on laboratory animal welfare and ethics, JiangSu Administration Office of Laboratory Animals. The animal experimentation was approved by the Committee of Laboratory Animal Welfare and Ethics, JiangSu Administration Office of Laboratory Animal, with the approval No. SYXK2008-00045.

### Animals and experimental design

Four multiparous Holstein cows (460 ± 16.4 kg body weight; 84 ± 25 days in milk at the beginning of the trial), fitted with 10-cm ruminal cannulas, were used in this experiment. The cows were assigned randomly to experimental treatments in a 2×2 crossover design trial. The treatments were a control diet (40% concentrate feed on a dry matter basis; control diet, COD) and a SARA induction diet (SARA induction diet, SAID; 70% concentrate feed on a dry matter (DM) basis) (Additional file 7: Table S3). The diets were formulated (NRC, 2001) to meet or exceed the energy requirements (18 kg/d dry matter intake) of a Holstein cow yielding 20 kg of milk/d with 3.50% milk fat and 3.10% true protein. The cows were fed at 0700 and 1800 h (one-half of the daily feed allowance at each feeding). Each experimental period consisted of 21 days. During the first 2 day, the level of dietary concentrate was increased gradually in the SAID group (by approximately 10 percentage units/d compared with COD). Dairy cows were offered feed ad libitum (approximately 5% orts). The cows had free access to fresh water during the trial.

### Sampling and measurements

Samples of ruminal fluid were taken every 2 h, starting from 0800, until 2000 to investigate the diurnal responses of pH on d 21 of the experimental period. To evaluate the effect of treatment on the risk of SARA, the duration for which the rumen pH was <5.8 from 0 to 12 h post-feeding was estimated. Relationship between ruminal pH and diet was modeled in function of time following feed distribution. For this model, a quadratic time term was added to the model to take into account the non-linear relationship between pH and postprandial time. Fecal samples were collected per rectum on d 12, 15, 17 and 21 before the morning feeding. Shortly after collection, 5 g of fresh feces was dissolved with 5 mL of water. The fecal pH was estimated using a glass electrode. A portion of the extract was centrifuged at 2000 *g*, and the supernatant was frozen at −20°C until analysis of VFA, which was completed within two weeks after sample collection [40]. The remaining fecal samples were sealed in sterile polypropylene containers, and were frozen at −80°C prior to deoxyribonucleic acid (DNA) extraction and starch analysis.

### DNA isolation

Feces collected on d 21 was used to extract DNA according to a bead-beating method using a mini-bead beater (Biospec Products, USA), followed by phenol–chloroform extraction [40]. The solution was precipitated with ethanol and the pellets were suspended in 50 μl of Tris-EDTA buffer. The DNA samples were quantified using a Nanodrop spectrophotometer (Nyxor Biotech, Paris, France).

### DNA pyrosequencing

The universal 16S rRNA gene primers (*Escherichia coli* positions 8 to 533: E8F 5^′^-AGA GTT TGA TCC TGG CTC AG-3^′^ and E533R 5^′^-TTA CCG CGG CTG CTG GCA C-3^′^) were chosen for the amplification and subsequent pyrosequencing of the polymerase chain reaction (PCR) products. The PCR mixture (final volume, 50 μl) contained 10 μl 5-fold reaction buffer (TransStart™ FastPfu Buffer, TransGen Biotech), <100 ng of DNA, 0.4 μM each primer, 0.5 U Pfu polymerase (TransStart™ FastPfu DNA Polymerase, TransGen Biotech), and 2.5 mM deoxyribonucleoside triphosphate. For each sample, three independent PCRs were performed using a MG96+Thermal Cycler (LongGene Scientific Instruments Co., Ltd). The PCR conditions were as follows: 95°C for 3 min; 25 cycles of denaturation (95°C; 0.5 min), annealing (52°C; 0.5 min), and extension (72°C; 0.5 min); followed by the final elongation (72°C; 10 min). The DNA was quantified using a TBS-380 Mini-Fluorometer (Promega Corporation, CA, USA). The sequences of the partial 16S rRNA genes were determined by using a Roche 454 FLX Pyrosequencer (Roche, Mannheim, Germany). Amplicons were sequenced as recommended in the instructions of the manufacturer for amplicon sequencing. The end fragments were blunted and tagged on both ends with one of eight ligation adaptors that contained a unique 10-bp sequence and a short four-nucleotide sequence (TCAG) called a sequencing key, which were recognized by the system software and the priming sequences.

### Analysis of pyrosequencing-derived data

The primer sequences were excluded after alignment, and sequences that were shorter than 200 bp in length or of low quality were removed from the pyrosequencing-derived data sets. All the analyses were performed using the MOTHUR program (http://www.mothur.org). Rarefaction analysis was also performed using the MOTHUR program. For taxonomy-based analysis, the SILVA database project (http://www.arb-silva.de) was used as a repository for the aligned rRNA sequences.

### Multivariate statistics

Double dendograms were constructed using the comparative functions and multivariate hierarchical clustering methods of NCSS 2007 (NCSS, Kaysville, Utah), on the basis of the abundances of the bacterial groups at different taxonomic levels. Clustering was performed using the weighted pair linkage and Manhattan distance methods with no scaling. It should be noted that the dendogram linkages of the bacterial genera are not phylogenetic but are related to the abundance among samples. Clustering on the basis of diet was based similarly upon the relative abundances of bacterial groups at different taxonomic levels among individual samples.

### Data analysis

The ruminal pH, fecal pH and VFA data were analyzed with the MIXED procedure of SPSS (SPSS v. 16, SPSS Inc., Chicago, IL) according to the following model:

(1) $$Y_{\text{ij}}=\mu +D+T+\text{TD}+S+P+C_{j}+e_{\text{ij}}$$

Where Y_ij_=is the ith observation (the ruminal pH, fecal pH or specific VFA concentration in mmol/L) from the jth cow; μ=overall mean; D=fixed effect of diet, T=fixed effect of measurement time (l=1-4 for days, and 1–7 for hours), TD=fixed effect of diet by time interaction, S=fixed effect of group, P=fixed effect of period (1st or 2nd), C_j_=random cow effect, e_ij_=residual error for the ith observation from the jth cow, residual terms are assumed to follow normal distributions. Measurements collected at different times, on the same cow, were, therefore, considered as repeated measures in the ANOVA, whereby the effects of day and hour were evaluated as fixed effect in the model.

For bacterial abundance analysis, the feces collected on d 21 were used in the following pyrosequencing experiment, thus, the microbial data were analyzed using the GLM procedure of SPSS (SPSS v. 16, SPSS Inc., Chicago, IL), according to the model shown below:

(2) $$Y_{i}=µ+D+P+\text{DP}+e_{i}$$

Where Y_i_ is the observation (the relative abundance of a given bacterial phyla, class, order, family, or specie (in %)); μ is the overall mean; D is the fixed effect of diet; P is fixed effect of study period (1st or 2nd); DP is the interaction between treatment and study period, and e_i_ is the residual term for the ith observation; corrections of p-values for multiple testing were performed using Bonferroni tests. Residual terms are assumed to follow normal distributions. Significance was declared at *P* <0.05 and a tendency was considered at 0.05 <*P* < 0.10.

Correlations between each fecal VFA concentration and the abundance of the genera or species were assessed by Pearson’s correlation test using GraphPad Prism version 5.00 (GraphPad Software, San Diego, CA, USA). Significance was declared at *P* <0.05.

## Competing interests

The authors declare that they have no competing interests.

## Authors’ contributions

MSY and ZHWY designed the feeding trial which was conducted by ZHRY and WDS. MSY performed sequence and bioinformatics analysis. MSY analyzed and interpreted the data, and drafted the article. All authors provide editorial content and have read and approved the final manuscript.

## Supplementary Material

Additional file 1: Figure S1 Influence of SAID feeding on fecal microbiota of dairy cattle at the level of bacterial class. A. Distribution of all bacterial classes among diets and animals as revealed by heatmap. B. The bacterial classes for which abundance was significantly affected by the diet. Legend as for **Figure 2**. Click here for file

Additional file 2: Figure S2 Influence of SAID feeding on fecal microbiota of dairy cattle at the level of bacterial order. A. Distribution of all bacterial orders among diets and animals. B. The bacterial orders for which abundance was significantly affected by the diet. Legend as for **Figure 2**. Click here for file

Additional file 3: Figure S3 Influence of SAID feeding on fecal microbiota of dairy cattle at the level of bacterial family. A. Distribution of the top (≥ 99.8% abundant) families observed among dietary treatments. B. The bacterial families for which abundance was significantly affected by the diet. Legend as for **Figure 2**. Click here for file

Additional file 4: Figure S4 Venn diagram of the overlap between observed OTUs at 3% divergence in COD and SAID groups. The number of OTUs found exclusively in COD was 1349 and in SAID samples was 1207. The number of OTUs shared between COD and SAID was 440. The percentage of shared OTUs was 21%. Data are also represented by the phylum to which the detected OTUs belong. Data regarding genera are shown as supplementary material. Click here for file

Additional file 5: Table S1 The OTUs at the genus level in COD and SAID groups. Click here for file

Additional file 6: Table S2 The changes in fecal microbial composition at the species level. Only the species that were significantly affected in percentage by the type of diet are presented. Click here for file

Additional file 7: Table S3 Diet composition and ingredients of experimental diets. Click here for file
