# Supplementary material for: The diversity of the fecal bacterial community and its relationship with the concentration of volatile fatty acids in the feces during subacute rumen acidosis in dairy cows
Source: BMC Vet Res. 2012 Dec 6;8:237. doi: 10.1186/1746-6148-8-237 (PMC3582618; doi:10.1186/1746-6148-8-237)
Supplement: Additional file 1: Figure S1 — Influence of SAID feeding on fecal microbiota of dairy cattle at the level of bacterial class. A. Distribution of all bacterial classes among diets and animals as revealed by heatmap. B. The bacterial classes for which abundance was significantly affected by the diet. Legend as for Figure 2. [file 1746-6148-8-237-S1.doc]

**Figure S1 Influence of SAID feeding on fecal microbiota of dairy cattle at the level of bacterial class**. A. Distribution of all bacterial classes among diets and animals as revealed by heatmap. B. The bacterial classes for which abundance was significantly affected by the diet. Legend as for Figure 2.

A

Relative Abundance(%)

Diet

Distance

B
