# Supplementary material for: The diversity of the fecal bacterial community and its relationship with the concentration of volatile fatty acids in the feces during subacute rumen acidosis in dairy cows
Source: BMC Vet Res. 2012 Dec 6;8:237. doi: 10.1186/1746-6148-8-237 (PMC3582618; doi:10.1186/1746-6148-8-237)
Supplement: Additional file 2: Figure S2 — Influence of SAID feeding on fecal microbiota of dairy cattle at the level of bacterial order. A. Distribution of all bacterial orders among diets and animals. B. The bacterial orders for which abundance was significantly affected by the diet. Legend as for Figure 2. [file 1746-6148-8-237-S2.doc]

**Figure S2 Influence of SAID feeding on fecal microbiota of dairy cattle at the level of bacterial order**. **A**. Distribution of all bacterial orders among diets and animals. **B**. The bacterial orders for which abundance was significantly affected by the diet. Legend as for Figure 2.

A

Relative Abundance(%)

Diet

Distance

B
