# Supplementary material for: The diversity of the fecal bacterial community and its relationship with the concentration of volatile fatty acids in the feces during subacute rumen acidosis in dairy cows
Source: BMC Vet Res. 2012 Dec 6;8:237. doi: 10.1186/1746-6148-8-237 (PMC3582618; doi:10.1186/1746-6148-8-237)
Supplement: Additional file 3: Figure S3 — Influence of SAID feeding on fecal microbiota of dairy cattle at the level of bacterial family. A. Distribution of the top (≥ 99.8% abundant) families observed among dietary treatments. B. The bacterial families for which abundance was significantly affected by the diet. Legend as for Figure 2. [file 1746-6148-8-237-S3.doc]

F**igure S3 Influence of SAID feeding on fecal microbiota of dairy cattle at the level of bacterial family**. **A**. Distribution of the top (≥ 99.8% abundant) families observed among dietary treatments. **B**. The bacterial families for which abundance was significantly affected by the diet. Legend as for Figure 2.

A

Relative Abundance(%)

Diet

Distance

B
