# Supplementary material for: The diversity of the fecal bacterial community and its relationship with the concentration of volatile fatty acids in the feces during subacute rumen acidosis in dairy cows
Source: BMC Vet Res. 2012 Dec 6;8:237. doi: 10.1186/1746-6148-8-237 (PMC3582618; doi:10.1186/1746-6148-8-237)
Supplement: Additional file 4: Figure S4 — Venn diagram of the overlap between observed OTUs at 3% divergence in COD and SAID groups. The number of OTUs found exclusively in COD was 1349 and in SAID samples was 1207. The number of OTUs shared between COD and SAID was 440. The percentage of shared OTUs was 21%. Data are also represented by the phylum to which the detected OTUs belong. Data regarding genera are shown as supplementary material. [file 1746-6148-8-237-S4.doc]

**Figure S4** **Venn diagram of the overlap between observed OTUs at 3% divergence in COD and SAID groups**. The number of OTUs found exclusively in COD was 1349 and in SAID samples was 1207. The number of OTUs shared between COD and SAID was 440. The percentage of shared OTUs was 21%. Data are also represented by the phylum to which the detected OTUs belong. Data regarding genera are shown as supplementary material.
