# Supplementary material for: The diversity of the fecal bacterial community and its relationship with the concentration of volatile fatty acids in the feces during subacute rumen acidosis in dairy cows
Source: BMC Vet Res. 2012 Dec 6;8:237. doi: 10.1186/1746-6148-8-237 (PMC3582618; doi:10.1186/1746-6148-8-237)
Supplement: Additional file 5: Table S1 — The OTUs at the genus level in COD and SAID groups. [file 1746-6148-8-237-S5.doc]

**Table S1**  Diet composition and ingredients of experimental diets

| Ingredients (% of DM)^1^ | COD | SAID |
| --- | --- | --- |
| Alfalfa hay | 27.00 | 12.00 |
| Leymus chinensis | 3.00 | 4.00 |
| Straw | 0.00 | 4.00 |
| Maize silage | 30.00 | 10.00 |
| Maize | 20.00 | 45.00 |
| Wheat bran | 0.00 | 1.40 |
| Soybean meal | 10.85 | 15.00 |
| cottonseed meal | 6.00 | 5.00 |
| Calcium carbonate | 0.40 | 0.85 |
| Calcium hydrogen phosphate | 0.75 | 0.75 |
| Sodium bicarbonate | 0.75 | 0.75 |
| Sodium chloride | 0.50 | 0.50 |
| Dairy premix^2^ | 0.75 | 0.75 |
| Nutrient composition (% of DM) | | |
| NEL(Mcal/kg of DM) | 1.54 | 1.71 |
| Crude protein (CP) | 15.8 | 15.8 |
| Neutral detergent fiber (NDF) | 34.9 | 24.7 |
| Acid detergent fiber | 23.0 | 13.2 |
| Non-fiber carbohydrates (NFC)d | 36.1 | 46.3 |
| Calcium | 0.88 | 0.82 |
| Phosphorous | 0.51 | 0.53 |

^1^Fed as TMR.

^2^Contained Mn, 0.24%; K, 0.5%; S, 0.2%; Zn, 4,000 mg/kg; Cu, 1,000 mg/kg; Mn, 2,500 mg/kg; I, 64 mg/kg; Co, 5 mg/kg; vitamin A, 1,000,000 IU/kg; vitamin D, 110,000 IU/kg, and vitamin E, 6000,000 IU/kg.
