# Supplementary material for: The diversity of the fecal bacterial community and its relationship with the concentration of volatile fatty acids in the feces during subacute rumen acidosis in dairy cows
Source: BMC Vet Res. 2012 Dec 6;8:237. doi: 10.1186/1746-6148-8-237 (PMC3582618; doi:10.1186/1746-6148-8-237)
Supplement: Additional file 6: Table S2 — The changes in fecal microbial composition at the species level. Only the species that were significantly affected in percentage by the type of diet are presented. [file 1746-6148-8-237-S6.doc]

Table S2 The OTUs at the genus level in COD and SAID group

| *Genus* | Unquie OTU | | Shared OTU |
| --- | --- | --- | --- |
|  | COD | SAID |  |
| Unclassified *Ruminococcaceae* | 154 | 97 | 67 |
| Unclassified *Lachnospiraceae* | 85 | 95 | 61 |
| Unclassified *Rikenellaceae* | 50 | 9 | 12 |
| Unclassified *Clostridiales* | 47 | 28 | 20 |
| Unclassified Bacteria | 46 | 25 | 18 |
| Unclassified *Planococcaceae* | 41 | 7 | 9 |
| Unclassified *Bacteroidales* | 36 | 50 | 10 |
| Unclassified Firmicutes | 36 | 13 | 2 |
| *Acinetobacter* | 36 | 6 | 10 |
| *Turicibacter* | 28 | 17 | 17 |
| Unclassified *Mollicutes* | 27 | 39 | 18 |
| Unclassified *Peptostreptococcaceae* | 27 | 8 | 13 |
| *Lysinibacillus* | 25 | 3 | 5 |
| *Solibacillus* | 19 | 2 | 7 |
| *Clostridium* | 16 | 4 | 5 |
| *Ruminococcus* | 14 | 22 | 10 |
| Unclassified *Erysipelotrichaceae* | 13 | 14 | 8 |
| *Alistipes* | 13 | 2 | 4 |
| *Bacillus* | 12 | 9 | 15 |
| Unclassified *Enterobacteriaceae* | 11 | 12 | 12 |
| *Enterococcus* | 9 | 16 | 12 |
| *Butyrivibrio* | 8 | 33 | 11 |
| Unclassified *Prevotellaceae* | 8 | 8 | 5 |
| *Treponema* | 8 | 1 |  |
| *Kurthia* | 7 | 4 | 1 |
| *Mogibacterium* | 6 | 15 | 9 |
| *Paenibacillus* | 6 | 2 | 4 |
| Unclassified *Cyanobacteria* | 6 | 1 |  |
| Unclassified *Coriobacteriaceae* | 5 | 10 | 4 |
| *Bacteroides* | 5 | 8 | 6 |
| Unclassified *Bacilli* | 5 | 3 |  |
| *Corynebacterium* | 4 | 8 | 4 |
| *Stenotrophomonas* | 3 | 10 | 4 |
| Unclassified *Gammaproteobacteria* | 3 | 8 | 4 |
| *Thalassospira* | 3 | 1 |  |
| *Acetitomaculum* | 2 | 12 | 5 |
| Unclassified *Bacteroidetes* | 2 | 4 |  |
| *Anaerotruncus* | 2 | 3 | 1 |
| *Solobacterium* | 2 | 2 | 7 |
| *Phascolarctobacterium* | 2 | 1 |  |
| *Pseudobutyrivibrio* | 2 | 1 |  |
| *Streptococcus* | 2 | 1 |  |
| Unclassified *Lactobacillales* | 2 | 1 | 1 |
| Unclassified *Lentisphaerae* | 2 | 1 | 2 |
| Unclassified *Planctomycetaceae* | 2 | 1 | 1 |
| *Prevotella* | 1 | 19 | 1 |
| *Atopobium* | 1 | 4 | 6 |
| *Aeriscardovia* | 1 | 2 | 1 |
| Unclassified *Aerococcaceae* | 1 | 2 |  |
| *Pseudomonas* | 1 | 1 |  |
| Unclassified *Peptococcaceae* | 1 | 1 |  |
| Unclassified *Bacillales* | 10 |  |  |
| *Phocaeicola* | 5 |  |  |
| *Cellulosilyticum* | 4 |  |  |
| *Lactococcus* | 4 |  |  |
| *Akkermansia* | 2 |  |  |
| *Barnesiella* | 2 |  |  |
| *Brevibacillus* | 2 |  | 1 |
| *Nocardiopsis* | 2 |  | 1 |
| *Papillibacter* | 2 |  |  |
| *Planomicrobium* | 2 |  |  |
| *Spirochaeta* | 2 |  |  |
| *Actinomyces* | 1 |  |  |
| *Anaerofustis* | 1 |  |  |
| *Arthrobacter* | 1 |  |  |
| *Asteroleplasma* | 1 |  |  |
| *Carnobacterium* | 1 |  |  |
| *Delftia* | 1 |  |  |
| *Denitrobacterium* | 1 |  |  |
| *Desemzia* | 1 |  |  |
| *Dietzia* | 1 |  |  |
| *Enterobacter* | 1 |  |  |
| *Klebsiella* | 1 |  | 1 |
| *Legionella* | 1 |  |  |
| *Microbacterium* | 1 |  | 1 |
| *Nocardioides* | 1 |  |  |
| *Odoribacter* | 1 |  |  |
| *Paludibacter* | 1 |  |  |
| *Psychrobacter* | 1 |  |  |
| *Sharpea* | 1 |  | 1 |
| Unclassified *Acetobacteraceae* | 1 |  |  |
| Unclassified *Microbacteriaceae* | 1 |  |  |
| Unclassified *Moraxellaceae* | 1 |  |  |
| Unclassified *Promicromonosporaceae* | 1 |  |  |
| Unclassified *Pseudomonadales* | 1 |  |  |
| *Victivallis* | 1 |  |  |
| *Bifidobacterium* |  | 27 | 3 |
| *Subdoligranulum* |  | 12 |  |
| *Blautia* |  | 6 | 2 |
| *Succiniclasticum* |  | 4 | 1 |
| *Arcanobacterium* |  | 3 |  |
| *Coprococcus* |  | 3 | 2 |
| *Facklamia* |  | 3 |  |
| *Helcococcus* |  | 3 | 1 |
| Unclassified *Actinomycetales* |  | 3 |  |
| *Globicatella* |  | 2 |  |
| *Lactobacillus* |  | 2 | 1 |
| *Ochrobactrum* |  | 2 | 1 |
| *Rummeliibacillus* |  | 2 | 1 |
| *Selenomonas* |  | 2 |  |
| *Staphylococcus* |  | 2 |  |
| *Acholeplasma* |  | 1 |  |
| *Alkalibacter* |  | 1 |  |
| *Anaerococcus* |  | 1 |  |
| *Anaerofilum* |  | 1 |  |
| *Anaerostipes* |  | 1 |  |
| *Anaerovibrio* |  | 1 |  |
| *Campylobacter* |  | 1 |  |
| *Desulfovibrio* |  | 1 |  |
| *Faecalibacterium* |  | 1 |  |
| *Fastidiosipila* |  | 1 |  |
| *Halomonas* |  | 1 |  |
| *Helicobacter* |  | 1 |  |
| *Holdemania* |  | 1 |  |
| *Ignavigranum* |  | 1 |  |
| *Kocuria* |  | 1 |  |
| *Leucobacter* |  | 1 |  |
| *Mitsuokella* |  | 1 |  |
| *Moryella* |  | 1 |  |
| *Oligella* |  | 1 |  |
| *Oscillibacter* |  | 1 |  |
| *Oscillospira* |  | 1 |  |
| *Peptoniphilus* |  | 1 |  |
| *Petrimonas* |  | 1 |  |
| *Propionibacterium* |  | 1 |  |
| *Proteiniphilum* |  | 1 |  |
| *Pseudoramibacter* |  | 1 |  |
| *Roseburia* |  | 1 | 1 |
| *Succinivibrio* |  | 1 |  |
| *Sutterella* |  | 1 |  |
| *Syntrophococcus* |  | 1 | 1 |
| *Terribacillus* |  | 1 |  |
| Unclassified *Actinobacteria* |  | 1 |  |
| Unclassified *Alcaligenaceae* |  | 1 |  |
| Unclassified *Anaerolineaceae* |  | 1 |  |
| Unclassified *Bacillaceae* |  | 1 |  |
| Unclassified *Carnobacteriaceae* |  | 1 |  |
| Unclassified *Clostridia* |  | 1 |  |
| Unclassified *Flavobacteriaceae* |  | 1 |  |
| Unclassified *Myxococcales* |  | 1 |  |
| Unclassified *Porphyromonadaceae* |  | 1 |  |
| Unclassified *Proteobacteria* |  | 1 |  |
| Unclassified *Solirubrobacterales* |  | 1 |  |
| Unclassified *Xanthomonadaceae* |  | 1 |  |
| *Weissella* |  | 1 | 1 |
| *Yaniella* |  | 1 |  |
| *Acetobacter* |  |  | 1 |
| *Dorea* |  |  | 1 |
| *Enterorhabdus* |  |  | 1 |
| *Howardella* |  |  | 1 |
| *Parabacteroides* |  |  | 1 |
| *Paracoccus* |  |  | 1 |
| Unclassified *Rhizobiales* |  |  | 1 |
| Unclassified *Rickettsiales* |  |  | 1 |
| Total OTU | 909 | 767 | 440 |
