# Supplementary material for: The diversity of the fecal bacterial community and its relationship with the concentration of volatile fatty acids in the feces during subacute rumen acidosis in dairy cows
Source: BMC Vet Res. 2012 Dec 6;8:237. doi: 10.1186/1746-6148-8-237 (PMC3582618; doi:10.1186/1746-6148-8-237)
Supplement: Additional file 7: Table S3 — Diet composition and ingredients of experimental diets. [file 1746-6148-8-237-S7.doc]

**Table S3 The changes in fecal microbial composition at the species level**. Only the species that were significantly affected in percentage by the type of diet are presented.

| OTUs | COD(%) | SAID(%) | Std. Error | P value | Annotation | Phylum |
| --- | --- | --- | --- | --- | --- | --- |
| OTU1266 | 6.66 | 1.15 | 0.678 | 0.005 | S:*Solibacillus silvestris* | Firmicutes |
| OTU2022 | 4.67 | 0.74 | 0.353 | 0.001 | G:*Lysinibacillus* | Firmicutes |
| OTU2140 | 2.52 | 6.42 | 0.917 | 0.039 | G:*Turicibacter* | Firmicutes |
| OTU1695 | 1.08 | 5.41 | 0.765 | 0.016 | G:*Stenotrophomonas* | Proteobacteria |
| OTU8134 | 0.64 | 0.03 | 0.095 | 0.010 | G:*Clostridium* | Firmicutes |
| OTU6041 | 0.45 | 0.00 | 0.069 | 0.010 | F:*Ruminococcaceae* | Firmicutes |
| OTU1523 | 0.42 | 0.01 | 0.089 | 0.033 | Unclassified Bacteria | Unclassified Bacteria |
| OTU8091 | 0.40 | 0.14 | 0.052 | 0.024 | F:*Peptostreptococcaceae* | Firmicutes |
| OTU1398 | 0.37 | 0.05 | 0.022 | 0.001 | P:*Firmicutes* | Firmicutes |
| OTU1954 | 0.30 | 0.00 | 0.030 | 0.002 | S:*Kurthia gibsonii* | Firmicutes |
| OTU892 | 0.30 | 0.00 | 0.073 | 0.044 | G:*Acinetobacter* | Proteobacteria |
| OTU6070 | 0.23 | 0.00 | 0.018 | 0.001 | F:*Ruminococcaceae* | Firmicutes |
| OTU1295 | 0.20 | 0.01 | 0.023 | 0.004 | S:*Bacillus massiliensis* | Firmicutes |
| OTU2371 | 0.16 | 0.49 | 0.067 | 0.026 | G:*Turicibacter* | Firmicutes |
| OTU7571 | 0.16 | 0.01 | 0.012 | 0.001 | F:*Ruminococcaceae* | Firmicutes |
| OTU8143 | 0.11 | 1.10 | 0.184 | 0.019 | F:*Lachnospiraceae* | Firmicutes |
| OTU8577 | 0.11 | 0.02 | 0.022 | 0.045 | O:*Clostridiales* | Firmicutes |
| OTU1351 | 0.10 | 0.00 | 0.005 | 0.000 | G:*Lysinibacillus* | Firmicutes |
| OTU4279 | 0.10 | 0.00 | 0.023 | 0.038 | O:*Bacteroidales* | Bacteroidetes |
| OTU6196 | 0.10 | 0.24 | 0.026 | 0.019 | F:*Ruminococcaceae* | Firmicutes |
| OTU5143 | 0.09 | (0.00) | 0.018 | 0.020 | O:*Clostridiales* | Firmicutes |
| OTU1333 | 0.08 | 0.00 | 0.018 | 0.029 | G:*Solibacillus* | Firmicutes |
| OTU4856 | 0.08 | 0.00 | 0.015 | 0.016 | F:*Rikenellaceae* | Bacteroidetes |
| OTU1161 | 0.07 | 0.00 | 0.016 | 0.035 | S:*Acinetobacter schindleri* | Proteobacteria |
| OTU5962 | 0.07 | 0.01 | 0.005 | 0.001 | F:*Lachnospiraceae* | Firmicutes |
| OTU7239 | 0.06 | 0.00 | 0.009 | 0.007 | P:*Cyanobacteria* | Cyanobacteria |
| OTU4580 | 0.06 | 0.00 | 0.010 | 0.017 | O:*Bacteroidales* | Bacteroidetes |
| OTU6788 | 0.06 | 0.00 | 0.009 | 0.010 | F:*Ruminococcaceae* | Firmicutes |
| OTU8034 | 0.05 | 0.00 | 0.009 | 0.014 | F:*Ruminococcaceae* | Firmicutes |
| OTU8121 | 0.05 | 0.01 | 0.006 | 0.010 | O:*Clostridiales* | Firmicutes |
| OTU4912 | 0.05 | 0.00 | 0.005 | 0.003 | F:*Rikenellaceae* | Bacteroidetes |
| OTU7063 | 0.05 | 0.00 | 0.002 | 0.000 | F:*Lachnospiraceae* | Firmicutes |
| OTU8725 | 0.04 | 0.00 | 0.005 | 0.004 | G:*Thalassospira* | Proteobacteria |
| OTU1336 | 0.04 | 0.00 | 0.008 | 0.028 | F:*Planococcaceae* | Firmicutes |
| OTU7446 | 0.04 | 0.01 | 0.005 | 0.009 | F:*Ruminococcaceae* | Firmicutes |
| OTU2345 | 0.04 | 0.00 | 0.002 | 0.000 | G:*Lysinibacillus* | Firmicutes |
| OTU8179 | 0.04 | 0.01 | 0.003 | 0.013 | F:*Lachnospiraceae* | Firmicutes |
| OTU7135 | 0.04 | 0.00 | 0.004 | 0.005 | O:*Clostridiales* | Firmicutes |
| OTU4924 | 0.03 | 0.00 | 0.005 | 0.010 | F:*Rikenellaceae* | Bacteroidetes |
| OTU1159 | 0.02 | 0.13 | 0.023 | 0.034 | G:*Stenotrophomonas* | Proteobacteria |
| OTU1288 | 0.02 | 0.00 | 0.004 | 0.046 | F:*Planococcaceae* | Firmicutes |
| OTU1152 | 0.02 | 0.00 | 0.004 | 0.035 | O:*Pseudomonadales* | Proteobacteria |
| OTU430 | 0.02 | 0.00 | 0.004 | 0.035 | S:*Enterococcus casseliflavus* | Firmicutes |
| OTU7317 | 0.02 | (0.00) | 0.004 | 0.046 | F:*Peptostreptococcaceae* | Firmicutes |
| OTU4788 | 0.02 | 0.65 | 0.010 | 0.000 | S:*Bifidobacterium Pseudolongum* | Actinobacteria |
| OTU8188 | 0.02 | 0.00 | 0.004 | 0.032 | G:*Clostridium* | Firmicutes |
| OTU8389 | 0.02 | 0.00 | 0.003 | 0.020 | F:*Coriobacteriaceae* | Actinobacteria |
| OTU7665 | 0.02 | 0.00 | 0.003 | 0.020 | F:*Lachnospiraceae* | Firmicutes |
| OTU8196 | 0.02 | 0.00 | 0.003 | 0.020 | F:*Lachnospiraceae* | Firmicutes |
| OTU1389 | 0.02 | 0.00 | 0.003 | 0.020 | F:*Planococcaceae* | Firmicutes |
| OTU6568 | 0.02 | 0.00 | 0.003 | 0.020 | F:*Ruminococcaceae* | Firmicutes |
| OTU6941 | 0.02 | 0.00 | 0.003 | 0.020 | F:*Ruminococcaceae* | Firmicutes |
| OTU1367 | 0.02 | 0.00 | 0.003 | 0.020 | G:*Lysinibacillus* | Firmicutes |
| OTU267 | 0.01 | 0.00 | 0.000 | 0.000 | F:*Planococcaceae* | Firmicutes |
| OTU3850 | 0.01 | 0.00 | 0.000 | 0.000 | F:*Rikenellaceae* | Bacteroidetes |
| OTU6881 | 0.01 | 0.00 | 0.000 | 0.000 | F:*Ruminococcaceae* | Firmicutes |
| OTU3167 | 0.01 | 0.00 | 0.000 | 0.000 | G:*Bacteroides* | Bacteroidetes |
| OTU6161 | 0.01 | 0.00 | 0.001 | 0.000 | F:*Lachnospiraceae* | Firmicutes |
| OTU7613 | 0.01 | 0.00 | 0.001 | 0.000 | F:*Lachnospiraceae* | Firmicutes |
| OTU7015 | 0.01 | 0.00 | 0.001 | 0.000 | F:*Peptostreptococcaceae* | Firmicutes |
| OTU5577 | 0.01 | 0.00 | 0.001 | 0.000 | F:*Ruminococcaceae* | Firmicutes |
| OTU6049 | 0.01 | 0.00 | 0.001 | 0.000 | F:*Ruminococcaceae* | Firmicutes |
| OTU6687 | 0.01 | 0.00 | 0.001 | 0.000 | F:*Ruminococcaceae* | Firmicutes |
| OTU7507 | 0.01 | 0.00 | 0.001 | 0.000 | F:*Ruminococcaceae* | Firmicutes |
| OTU8018 | 0.01 | 0.00 | 0.001 | 0.000 | F:*Ruminococcaceae* | Firmicutes |
| OTU3596 | 0.01 | 0.00 | 0.001 | 0.000 | G:*Alistipes* | Bacteroidetes |
| OTU1346 | 0.01 | 0.00 | 0.001 | 0.000 | G:*Lysinibacillus* | Firmicutes |
| OTU4295 | 0.01 | 0.00 | 0.001 | 0.000 | G:*Phocaeicola* | Bacteroidetes |
| OTU1522 | 0.01 | 0.00 | 0.001 | 0.000 | G:*Turicibacter* | Firmicutes |
| OTU3053 | 0.01 | 0.00 | 0.001 | 0.000 | O:*Bacteroidales* | Bacteroidetes |
| OTU7116 | 0.01 | 0.00 | 0.001 | 0.000 | O:*Clostridiales* | Firmicutes |
| OTU7248 | 0.01 | 0.00 | 0.001 | 0.000 | O:*Clostridiale* | Firmicutes |
| OTU8833 | 0.01 | 0.00 | 0.001 | 0.000 | O:*Clostridiales* | Firmicutes |
| OTU3184 | 0.01 | 0.14 | 0.008 | 0.000 | O:*Bacteroidales* | Bacteroidetes |
| OTU3528 | 0.01 | 0.03 | 0.005 | 0.030 | G:*Solobacterium* | Firmicutes |
| OTU6299 | 0.01 | 0.08 | 0.004 | 0.000 | G:*Mogibacterium* | Firmicutes |
| OTU6120 | 0.01 | 0.06 | 0.006 | 0.004 | *Unclassified Bacteria* | Unclassified Bacteria |
| OTU8435 | 0.00 | 0.18 | 0.024 | 0.006 | G:*Subdoligranulum* | Firmicutes |
| OTU7746 | 0.00 | 0.05 | 0.008 | 0.009 | F:*Ruminococcaceae* | Firmicutes |
| OTU8040 | 0.00 | 0.05 | 0.005 | 0.002 | F:*Ruminococcaceae* | Firmicutes |
| OTU8171 | 0.00 | 0.04 | 0.006 | 0.009 | F:*Lachnospiraceae* | Firmicutes |
| OTU759 | 0.00 | 0.01 | 0.003 | 0.022 | F:*Enterococcaceae* | Firmicutes |
| OTU8528 | 0.00 | 0.01 | 0.003 | 0.022 | F:*Lachnospiraceae* | Firmicutes |
| OTU5880 | 0.00 | 0.01 | 0.003 | 0.022 | F:*Ruminococcaceae* | Firmicutes |
| OTU9102 | 0.00 | 0.01 | 0.003 | 0.022 | O:*Clostridiales* | Firmicutes |
| OTU5259 | 0.00 | 0.01 | 0.003 | 0.031 | G:*Butyrivibrio* | Firmicutes |
| OTU5301 | 0.00 | 0.01 | 0.003 | 0.031 | G:*Butyrivibrio* | Firmicutes |
| OTU8906 | (0.00) | 0.03 | 0.007 | 0.028 | F:*Coriobacteriaceae* | Actinobacteria |

S: = species; G: = genera; F: = family; O: = order; C: = class.

The OTUs were sorted on the basis of their relative abundance in the rumen microbial community of cattle fed with the control diet, in descending order.
